# Geographic variation of hysterectomy rates in the Israeli health care system during the years 2007–2016

**DOI:** 10.1186/s13584-019-0321-9

**Published:** 2019-07-16

**Authors:** Roy Lauterbach, Mendlovic Joseph, Ziona Haklai, Lavie Gil, Lior Lowenstein

**Affiliations:** 1 Department of Obstetrics and Gynecology, Rambam Health Care Campus, and Ruth and Bruce Rappaport Faculty of Medicine, Technion, Haifa, Israel; 2 Shaare Zedek Medical Center, Jerusalem, Israel; 3 Ministry of Health of Israel, Jerusalem, Israel; 4 Shaare Zedek Medical Center, Jerusalem, Israel; 5 Lady Davis Carmel Medical Center, Haifa, Israel

**Keywords:** Variation, Hysterectomy, Implementation, Technology

## Abstract

**Background:**

In 2014 the OECD published a report regarding inter-regional variation of hysterectomies in 13 countries including Israel. Variance in hospital admission rates were also reported. The Israeli Ministry of Health has set as one of its main goals the reduction in differences in health care, particularly between the country’s periphery and central regions. These variations may reflect differences in characteristics, resource allocation, and medical staff employment, expertise and training. The advances in technology in the last decades including laparoscopic and robotic surgeries and the variance in their implementation emphasize the great regional variance. The aim of this study was to examine hysterectomy trends in the past decade with emphasis on regional differences.

**Methods:**

The study is based on information maintained by the Israeli Ministry of Health and portrays the trend in hysterectomy rates as a factor of indication, surgical approach and length of hospitalization as collected from the years 2007–2016.

**Results:**

Inter-regional significant differences were found between the 7 regions of Israel, though there was a clear trend toward a national 11–24% decrease in hysterectomy rates. A 2–4 time increase in laparoscopic hysterectomies was observed. There was a clear country-wide trend toward shortening hospital stay from 5 to 4 days in total.

**Conclusions:**

Hysterectomy rates have declined in the past decade due to the implementation of new technologies allowing earlier diagnosis and minimally invasive surgery on top of offering alternative, non-surgical treatment modalities. Uneven allocation of resources and manpower allowing technology implementation and optimal medical services may have contributed to the findings.

## Introduction

The OECD published a report in September 2014 regarding the inter-regional variation of various medical services during the years 2000–2011 in 13 countries, including Israel [1]. Inter-regional differences were found within several countries in the rates of certain procedures, including percutaneous transluminal coronary angioplasty, cardiac bypass surgery, hysterectomy and knee joint replacement surgery. In addition, higher rates of hospital admissions were observed in the peripheral regions of some countries [1]. Variation in medical treatment between regions in Israel has received increasing attention in recent years. The Israeli Ministry of Health has set as one of its main goals a reduction in differences in health care, particularly between the country’s periphery and central regions.

Variations in medical treatment and in the use of health services have been shown to reflect differences in characteristics of geographic regions [2–4]. Such characteristics include resource allocation, and medical staff employment, expertise and training.

A low level of inter-regional variation is generally presumed to indicate optimal medical services. However, this optimization is harder to achieve in regard to medical conditions that have a challenging course of diagnosis and treatment such as cancer, in contrast to fractures [5–7].

The last two decades have witnessed great advancements in surgical technology in general, and in the field of gynecology, specifically. Two examples of these technological advancements are robotic and laparoscopic surgeries, which have been implemented rapidly for both benign and oncological indications in Israel. These technologies allow shorter hospitalizations, faster recovery periods, fewer surgical complications and an expedited return to regular activity.

Indications for hysterectomy include pelvic organ prolapse, menstrual bleeding disorders, large myomatous uteri, uncontained bleeding during Cesarean Section and gynecological malignancies.

Between 7800 and 8000 hysterectomies are performed annually in public hospitals in Israel.

Variance between regions of the country in the implementation of these new technologies may result from differences in the availability of equipment and surgical skills.

The aim of this study was to examine trends in the past decade (2007–2016) in the rate of hysterectomies among Israeli women, with an emphasis on differences between regions, surgical indications, surgical approaches and patients` mean age.

## Materials and methods

This study presents data that was obtained from the records of the National Hospital Discharges Database (NHDD) maintained by the Israeli Ministry of Health. This database is continuously updated based on quarterly electronic reports from all acute care hospitals in Israel; and contains records of individual admissions, including demographic characteristics, surgical approach and length of hospitalization.

Database was searched according to ICD-9 codes for gynecological indications for hysterectomy including all of the following: malignant indication codes – 1821, m8381/3, m8381/0, m8381/1, m8380/0, m8380/1, m8380/3, 2331, 23,339, 23,330, 1809, 2190, 1838, 1839, 220, 1830, 1833, 1808, 1800, 1801, 1832, 1834, 1835, m9090/3, 1828, 179,62,132, 62,133, 62,130, 62,135, m8930/3; benign indication codes – 6271, r62382, 6170, 2189, 6181, r61814, r61811, r61812, r61813, 65,413, 65,412, 65,414, 65,411, 65,410, 2198, 2199, 2332, 6212, 62,134, 62,130, 2182, 2180, 2181, m8890/0, m8995/0, m8900/0, 61,884, 6189, 61,889, 6185, 61,809, 61,800, 6183, 6182, 6184, 6270, 6266.

Hysterectomy rates are presented for each of the seven districts in Israel, as defined by the Ministry of Interior. The procedures’ data, which was stratified by Israel’s 7 districts, was determined by patients’ residence, and not by the location of the medical facility in which the surgery took place, since it is not uncommon for medical services to be provided in districts other than the district of residence. Non-residents such as tourists were excluded from the analysis.

Hysterectomy rates were calculated and generally presented as rates per 100,000 women. We examined hysterectomy rates according to patient age (divided to ages 25–34, 35–44, 45–54, 55–64, 65–74, 75 &over), surgical indication (benign or malignant) and surgical approach (abdominal, vaginal or laparoscopic) with no regard to patient race (Jews and Arabs). Chi square of association was used for comparison between categorical parameters. Ten-year hysterectomy trend analysis was performed using the Joinpoint trend analysis software. Significance was determined for *P* value < 0.05.

## Results

The age-adjusted hysterectomy rate per 100,000 women aged 25 years and over decreased by 13.8%, from 261 in 2007 to 226 in 2016, with an annual percent change (APC) of 1.87% (*P* < 0.0001).

The highest hysterectomy rate was found among women aged 45–54 years. For this age group, the rate declined from 502 to 372 per 100,000 from 2007 to 2016, a decrease of 25.9% (APC-2.95%) (*P* < 0.0001).

**Fig. 1:**
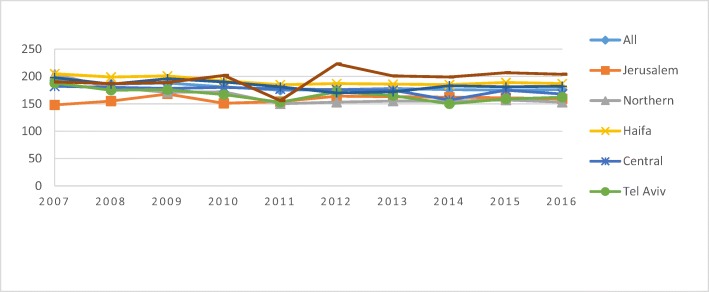
Overall hysterectomy age-adjusted rate per 100,000 females by district, 2007–2015

Figure 1 shows the changes in the hysterectomy rate between 2007 and 2016. District information is displayed in Table 1. The age-adjusted rate decreased by 24% (APC- 2.76%) (*P* < 0.0001) in the northern region of Israel compared to an 12% decrease (APC-1.68%) in the Haifa region (*P* = 0.04), the Tel-Aviv region (*P* = 0.002) and the southern region (*P* = 0.04). The rate increased by 10–12% in both the Jerusalem region (144.7 to 159.5) and the Judaea and Samaria region (184.3 to 206.9) (APCs-0.77 and 1.16%, respectively). Neither of the for mentioned changes proved to be statistically significant. In the beginning of the study period, hysterectomy rates were lower in Jerusalem than in other regions of the country (*P* < 0.001). In contrast, for the Judea and Samaria region, the rate was similar to the average rate in the beginning of the examined period, yet significantly higher at the end of the period (*P* = 0.01).

**Table 1:** District Characteristics

District	Population	Area (square kilometer)	Citizens per square kilometer	Number of hospitals in district
North	1,425,700	4473	319	7
Haifa	1,013,900	864	1174	4
Tel Aviv	1,406,400	176	7991	3
Center	2,157,500	1293	1669	6
Jerusalem	1,108,900	653	1698	2
South	1,272,100	14,185	90	3
Judaea and Samaria	413,400	5790	72	0

The duration of hospitalization following hysterectomy also decreased, from a mean of 5.2 days in 2007 to 4.3 days in 2016 (APC-1.88%), with the most prominent change occurring in the northern region of Israel, from 5.9 to 4.6 days (APC-2.45%).

**Fig. 2:**
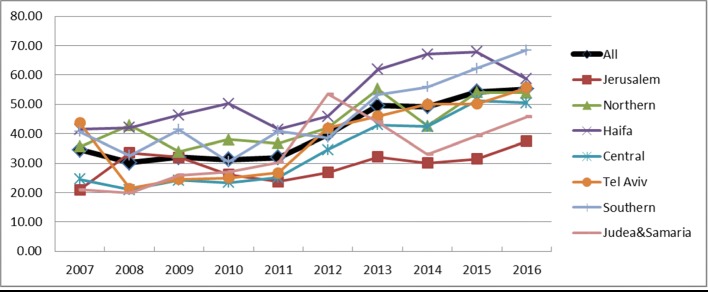
Laparoscopic hysterectomy age-adjusted rate per 100,000 females by district, 2007–2016

**Fig. 3:**
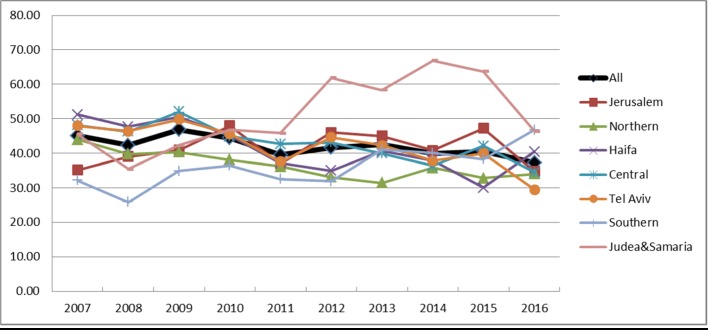
Vaginal hysterectomy age-adjusted rate per 100,000 females by district, 2007–2016

The hysterectomy approach changed significantly during the study period. While 60% of all hysterectomies in 2007 were abdominal, by 2016, only 48% were abdominal)APC-2.2%) (Fig. 2). The proportion of procedures performed laparoscopically increased in parallel from 18 to 31% (APC-5.58%) (Fig. 2). The vaginal approach remained steady at 25% (Fig. 3). The increase in laparoscopic hysterectomies reached statistical significance in all the regions of Israel. In Tel Aviv, the rate of laparoscopic hysterectomies was relatively high in 2007 (43.7 per 100,000) and was similar to the national average (54.3) in 2016 (APC-2.19%). Though a significant rise in laparoscopic hysterectomies was observed in the Jerusalem and the Judaea and Samaria region, the rates were significantly lower than in the other regions. The increased rate of laparoscopic hysterectomy was mainly attributed to the increased rate of endometrial carcinoma cases treated with endoscopic surgery which increased from 19% in 2007 to 30% in 2016 (APC-4.67%).

Analysis of surgical indications demonstrates that the rate of abdominal hysterectomy for benign indications remained stable during the years 2007–2016, around 55%. A small decrease was noted in the proportion of procedures for the treatment of uterine fibroids. The rate of abdominal hysterectomy for malignant indications remained stable during the years 2007–2016, around 40%. A small but prominent decrease was noted in the proportion of procedures for the treatment of uterine malignancies. With regard to the vaginal surgical approach, a rise in surgery rates was observed for benign indications, mainly pelvic organ prolapses, from 78 to 89% (APC-1.33). The rate of vaginal hysterectomy for malignant indications dropped from 8 to 3% over the study period (APC-9.34%).

The rate of laparoscopic hysterectomy for benign indications declined between 2007 and 2016 from 61 to 49%, while the rate for malignant indications rose from 32 to 45% (APCs-2.16 and 3.46%, respectively).

## Discussion

Hysterectomy is a common and major surgical procedure that may provide relief for a number of gynecological problems. Nonetheless, this procedure is often associated with negative health impacts. Previous studies indicate that hysterectomy rates and trends vary widely both within and between countries [1]; yet little is known about hysterectomy patterns in Israel.

Our findings demonstrate a significant and steady decline in hysterectomy rates over the last 10 years. The findings presented are comparable to those of studies that were conducted in Australia, Canada, the UK and the USA, and that demonstrated a reduction in the rate of hysterectomy over the last 20 years, perhaps due to the use of alternative expectant treatments [8–12]. A consistently higher rate was documented in the aforementioned countries among women living in rural compared to urban regions, and a strong inverse correlation between an area’s socioeconomic status and the hysterectomy rate [1]. This difference was not demonstrable in Israel due to the similarity across regions in terms of rurality and socioeconomic status and the lack of information regarding intra-regional differences between cities and rural areas.

Similar to our findings, the rate of hysterectomy in Canada decreased during 2003–2010, with substantial differences between provinces; and in some cases, prominent variation within the same province. The Canadian Society of Obstetricians and Gynecologists developed and updated clinical practice guidelines on the performance of hysterectomy. Indications for hysterectomy include: symptomatic fibroids, in which case the procedure provides a long-term solution for menorrhagia and pressure symptoms caused by the enlarged uterus; abnormal uterine bleeding after exclusion of treatable pathologies or after exhausting all medical alternatives; severe symptoms of endometriosis with previous treatment failure when fertility is no longer desired; pelvic pain associated with dysmenorrhea or significant pelvic disease with previous treatment failure; endometrial hyperplasia with atypia; non-invasive cervical adenocarcinoma in situ; staging of endometrial carcinoma, as well as of cervical, epithelial ovarian, and fallopian tube carcinoma; intractable postpartum hemorrhage with conservative therapy failure and uncontrolled bleeding; ruptured tubo-ovarian abscesses that is unresponsive to antibiotics; and acute menorrhagia refractory to medical and surgical treatment [13] . Though the abovementioned guidelines might have contributed to the reduction in hysterectomy rates overall, they have not reduced variations across provinces in Canada [1].

A previous study performed in the province of Quebec in the 1980s showed a consistent decline in the rate of hysterectomies for conditions including leiomyoma, endometriosis, disorders of menstruation and prolapse. A similar decline was observed in the current study.

In the current study, the highest hysterectomy rate was found among women aged 45–54 years. The Canadian study showed a consistent bimodal pattern, with a first peak occurring among women aged 40 to 44 years, and a second peak among women aged 65 to 69. The rate of hysterectomy was highest among women aged 45–54 years and remained at a high and steady level for women aged 55–74 years. No such pattern was evident in our findings of hysterectomies in Israel. A possible explanation for the difference in patterns between the studies is the small geographic size of Israel and denser population, with a greater number of medical centers per square feet, thus increasing the accessibility of gynecological follow-up. Another possible explanation is the well-documented high prevalence of epithelial ovarian cancer among Ashkenazi Jews, which may manifest in larger surgical volumes 10 years before normal prevalence, around 50 years of age.

In France, the total rate of hysterectomies decreased between 2005 and 2011, by approximately 20%; a 2-fold difference was observed between regions with the highest and lowest rates [1].

The findings reported herein concur with those of a study conducted in Australia, which showed a decline by 10% in total hysterectomy rate between the years 2000 and 2005 [10]. A 19% decline in the rate of abdominal hysterectomy was observed during that period, with a concurrent rise in laparoscopic surgeries. Similar to the findings of the current study, the highest rate of hysterectomy was for women aged 45–54 years. Indications for surgery were similar between that and the current study.

As was expected, with the implementation of the laparoscopic and robotic approach for hysterectomy, the rate of abdominal hysterectomy decreased significantly. This trend was also associated with a significant decrease in the length of hospitalization stay following the surgery. Our results show an uneven rate of implementation between the 7 districts in the country, which persisted over the years of the study. This variance may be due to longer implementation periods consequent to smaller surgical volumes and the religious characteristics of the populations in the 2 regions; the latter may have increased a preference for expectant over surgical management. In addition, variance between regions was observed in the total number of hysterectomies performed, with a higher rate in the rural and central regions and a lower rate in the regions in which a large religious population resides.

The rate of vaginal hysterectomy in Israel did not change during the 10-year study period, concurring with data from other countries [1, 8–12]; and supporting the notion that the rise in laparoscopic hysterectomies was on account of the decreased performance of abdominal hysterectomy. In the last five years, laparoscopic hysterectomy for the removal of myomatous uteri has decreased due to the decrease in morcellator use on account of possible dissemination of uterine sarcomas not diagnosed pre-op.

In regard to length of hospitalization, the results demonstrate a steady and prominent decrease, which reflects the advancement in surgical technology in the field of gynecology. Contrasting with this finding, the length of hospitalization among patients admitted in internal medicine units showed no decline.

## Conclusions

This study highlights factors that may affect rates of hysterectomy. Of particular interest is the modest influence of geographic region; this may reflect differences in socioeconomic status, parity and religious beliefs. The cohort is representative of the entire study population, thus enabling extrapolation of these findings.

A limitation of the study is that when comparing Israel to Canada and Australia, it is important to comment that Israel is of small size and patients may have their procedure performed all over the country since geographical distances are small. Thus, all Israeli citizens, wherever they live, have accessibility to modern endoscopic techniques.

Though the population is becoming older, the overall rate of hysterectomy is decreasing. This may be due in large part to the availability of alternative treatments, such as endometrial ablation, uterine artery embolization, and conservation of the uterus at the time of prolapse surgery repair. Also, due to new technologies assimilated in the field of gynecology and oncology, physicians` ability to diagnose treatable pathologies at an earlier stage may affect the rate of hysterectomies and surgical approach.

The public health impact of this study is that it underlines differences in medical approach and possibly in the quality of treatment provided between regions of the same country. This indicates that the provision of equal and optimal treatment among all citizens of a single country is far from realization.

## Policy implications

We cite the following issues as relevant to health care system policies:

1. Enhancement of health insurance surveillance in districts and regions that show distinct trends in hysterectomy rates (In Israel, we refer to the Jerusalem and Samaria regions), with emphasis on the difference in hysterectomy rates performed in the private sector in oppose to those performed in the public sector.
2. Investigation of explanations for differences between regions in rates of laparoscopic compared to abdominal and vaginal approaches, with an emphasis on the effect of private medical insurance rates.

## Data Availability

Availability of data and material-data is available in the national health ministry database.
